# Genetic variation within *IL18* is associated with insulin levels, insulin resistance and postprandial measures^☆^

**DOI:** 10.1016/j.numecd.2009.12.004

**Published:** 2011-07

**Authors:** M.C. Smart, G. Dedoussis, N. Yiannakouris, M.L. Grisoni, G.K. Dror, M. Yannakoulia, C. Papoutsakis, E. Louizou, C.S. Mantzoros, L. Melistas, M.D. Kontogianni, J.A. Cooper, S.E. Humphries, P.J. Talmud, on behalf of the EARSII Consortium

**Affiliations:** a Division of Cardiovascular Genetics, British Heart Foundation Laboratories, Department of Medicine, Royal Free and UCL Medical School, London, UK; b Department of Nutrition and Dietetics, Harokopio University, Athens, Greece; c Department of Home Economics and Ecology, Harokopio University, Athens, Greece; d INSERM, UMR_S 937, F-75013, Paris, France; e UPMC Univ Paris 06, UMR_S 937, F-75013, Paris, France; f Division of Endocrinology, Diabetes and Metabolism, Department of Medicine, Beth Israel Deaconess Medical Center (BIDMC), Harvard Medical School, Boston, MA, USA

**Keywords:** Interleukin 18, Obesity, Insulin resistance, Single nucleotide polymorphisms, Genetic variants, Haplotypes, AUC, area under the curve, CATAMERI, Catanzaro Metabolic Risk, CVD, cardiovascular disease, CI, confidence intervals, CHD, coronary heart disease, EARSII, European Atherosclerosis Research case control Study, FDR, false discovery rate, GENDAI, Gene-Diet Attica Investigation on childhood obesity, GrOW, Greek Obese Women, HWE, Hardy–Weinberg equilibrium, HOMA, homeostasis model assessment, IIPGA, Innate Immunity PGA, IR, insulin resistance, IL-18, Interleukin 18, LD, linkage disequilibrium, MI, myocardial infarct, MAF, minor allele frequency, OFTT, oral fat tolerance test, OGTT, oral glucose tolerance test, QUICKI, quantitative insulin sensitivity check index, SNP, single nucleotide polymorphism, tSNPs, tagging single nucleotide polymorphisms, T2D, type 2 diabetes, UTR, untranslated region

## Abstract

**Background and aims:**

IL-18 expression is up-regulated in atherosclerotic plaques, and higher levels are seen in obese and Type 2 Diabetic individuals. More recently, a possible role for IL-18 in glucose and energy homeostasis has been suggested.

**Methods and results:**

We investigated variation within the *IL18* gene and its association with measures of obesity and the metabolic syndrome. Five *IL18* tagging single nucleotide polymorphisms (rs1946519, rs2043055, rs549908, rs360729, rs3882891) were selected and genotyped in the Gene-Diet Attica Investigation on childhood obesity (GENDAI) (age range 10–14 yrs); in young European men in the second European Atherosclerosis Research offspring Study (EARSII), an offspring study (age range 18–28 yrs) and in a group of healthy women from the Greek Obese Women study (GrOW) (age range 18–74 yrs). Six common haplotypes were observed. In GrOW, Hap6 (Frequency-2.6%) was associated with higher insulin levels (*p* < 0.0001), estimates of HOMA_-Insulin Resistance_ (*p* < 0.0001) and HOMA_-β-cell_ (*p* < 0.0001) compared to the common haplotype Hap1 (Frequency-33.2%). In EARSII, rs2043055 was associated with peak and area under the curve triglycerides (*p* = 0.001 and *p* = 0.002, respectively) after an oral fat tolerance test in ‘cases’ but not ‘controls’. None of the haplotypes were associated with measures of body fatness in any of the studies.

**Conclusion:**

Association of *IL18* variation with insulin levels and estimates of insulin resistance were only observed in our adult study, suggesting that the effects of IL-18 are only associated with increasing age. Taken together with the association of *IL18* variants with post-prandial measures, this provides support for IL-18 as a metabolic factor.

## Introduction

Interleukin-18 (IL-18), a pro-inflammatory cytokine produced by macrophages, is involved in both adaptive and innate immune responses [1]. IL-18 stimulates interferon-γ production in T-lymphocytes and natural killer cells, both of which play a role in atherosclerotic progression [2]. IL-18 expression is up-regulated in atherosclerotic plaques and associated with the presence of pathological signs of plaque instability [3]. IL-18 levels have since been confirmed as an independent predictor of coronary events in healthy middle aged men [4].

More recently IL-18 has been suggested to be an adipogenic cytokine [5], associated with excess adiposity [6]. Adipocytes from obese individuals produce higher levels of IL-18 compared to lean individuals [7] and higher circulating IL-18 levels were observed in obese individuals [8], and those with Type 2 Diabetes (T2D) and the metabolic syndrome [9]. Several studies have suggested that muscle is the major source of circulating IL-18 in humans, and not adipocytes [10,11]. Nevertheless, IL-18 levels have been have been consistently associated with insulin resistance measured by the homeostasis model assessment (HOMA) [12] and studies in humans [13] and *il18* ^−/−^ mice [14] suggest a possible role for IL-18 in insulin sensitivity and energy homeostasis.

Variation in *IL18* has been associated with IL-18 levels and measures of obesity in men with T2D and subjects with advanced coronary heart disease [15] and with insulin sensitivity in the Catanzaro Metabolic Risk (CATAMERI) study in Italy [16]. We sought to investigate the association of IL-18 gene variants with measures of obesity and the metabolic syndrome in different age ranges; in healthy children who participated in the Gene – Diet Attica Investigation on childhood obesity (GENDAI) (aged 10–14 years) and a group of healthy women from the Greek Obese Women study (GrOW) (aged 18–74 years). We also examined the effect of these *IL18* variants in response to an oral fat tolerance test (OFTT) and an oral glucose tolerance test (OGTT) in young men (aged 18–28 years) in the second European Atherosclerosis Research Study (EARSII), an offspring study of ‘cases’ with a paternal history of premature coronary heart disease (CHD) with matched ‘controls’.

## Methods

### Study populations

#### Gene – Diet Attica Investigation on childhood obesity (GENDAI)

Subjects were recruited from public schools in the Attica region of Greece and a total of 1138 children were enrolled. Due to the heterogeneity in allele frequencies between Greek and non-Greek Caucasians, only children of Greek nationality (mean age: 11.2 ± 0.7 years; *n* = 882; 418 males and 464 females) were included in the present study. Details of recruitment, body composition assessment and biochemical analysis have been previously described [17]. Parents or guardians and participating children gave their informed consent prior to inclusion in the study. The study was approved by the Institutional Review Board of Harokopio University and the Greek Ministry of Education.

#### European Atherosclerosis Research case control Study (EARSII)

Subjects were recruited from 14 European university student populations, men aged 18–28 years, from 11 European countries. The countries were divided into four regions: Baltic (Estonia and Finland); United Kingdom; Middle Europe (Belgium, Denmark, Germany, and Switzerland); and South Europe (Greece, Italy, Portugal, and Spain). The study comprises ‘Cases’, classified on the basis of their father having an early myocardial infarct (MI) (pre-55 years; *n* = 407) and age-matched controls (*n* = 415). Each participant was administered a standard OGTT (100 mg) and a standardised OFTT (1493Kcal) after a 12-h overnight fast. Venous blood samples were drawn at 0, 30, 60, 90, and 120 min after OGTT for determination of insulin and glucose concentrations and at 0, 2, 3, 4 and 6 h after OFTT for triglyceride concentrations. Details of these assays have been reported previously [18].

#### Greek Obese Women Study (GrOW)

A total of 379 women of Greek origin without a known history of diabetes, cardiovascular disease or cancer were enrolled in this study, which was approved by the Institutional Review Board of Harokopio University. All participants gave their informed consent. Details of body composition assessment and biochemical analysis have been previously described [19]. Fasting glucose concentrations >126 mg/dL, cortisol treatment and lipid lowering medication were criteria for exclusion from the analysis. Thus, our analysis was restricted to 349 apparently healthy women without diabetes or cardiovascular disease (CVD).

### Experimental procedures

#### Tagging single nucleotide polymorphism identification

There are two known functional variants within the promoter (rs187238) and 3′ untranslated region (UTR) (rs5744292) of the *IL18* gene [16], however, we wanted to capture as much of the variation across the *IL18* gene as possible. Therefore, a tagging single nucleotide polymorphism (tSNP) set comprising variants −9731 G > T, −5848 T > C, +4860A > C, +8855 T > A, and +11015 T > G (rs1946519, rs2043055, rs549908, rs360729, rs3882891, respectively) was selected, based on haplotypes derived from the Innate Immunity PGA (IIPGA) Caucasian re-sequencing data (http://innateimmunity.net). The set was estimated to capture more than 90% of variation within the 21-kilobase *IL18* region, stretching from 1 kilobase upstream to 300 base pairs downstream of the gene. The set comprises three intronic variants (rs2043055, rs360729, rs3882891), a proximal promoter variant (rs1946519), and one synonymous single nucleotide polymorphism (SNP) (rs549908) within exon 4 which have been previously studied [15].

#### IL18 tSNP genotyping

All five tSNPs were genotyped using TaqMan technology and probes designed by Applied Biosciences (ABI, Warrington UK). Fluorescence was measured with the ABI Prism 7900HT detection system analysed with the ABI TaqMan 7900HT v3.1software. Primers and MGB probes are available upon request.

#### Insulin resistance and β-cell functionalism calculation

β-cell function and insulin resistance (IR) estimates were derived using HOMA with the following formula: HOMA_-IR_ = fasting insulin (μIU/ml) × fasting glucose (mmol/l)/22.5 [20], HOMA-_β-cell_ = fasting insulin (μIU/ml) × 20/fasting glucose (mmol/l) − 3.5 [21], quantitative insulin sensitivity check index (QUICKI) = 1/(log(fasting insulin (μIU/ml)) + log(fasting glucose (mg/dl)) [22].

#### Statistical methods

The majority of statistical analyses were performed using Intercooled Stata 10.2 for Windows (StataCorp LP, USA). A χ^2^ test compared observed numbers of each genotype with those expected for a population in Hardy-Weinberg equilibrium (HWE). Data were transformed, when necessary to approximate a normal distribution. tSNPs were first analysed individually for association with baseline and post-prandial measures. Linear regressions were used for association analyses. Covariates were established using a backwards stepwise regression. Covariates for GENDAI included; height, age, gender, BMI and mean Tanner score. Covariates for EARSII included; BMI, smoking, age, region, and fasting levels when analysing post-prandial data. Covariates for GrOW included; age, estrogen use, smoking status, menopausal status and body fat %. *P* values less than 0.01 were considered significant. For the univariate analyses, setting a threshold of significance was the chosen method above Bonferroni corrections. Linkage disequilibrium (LD) between sites was estimated in Stata with the pairwise Lewontin's D' and r^2^ using the *pwld* function (http://www-gene.cimr.cam.ac.uk/clayton/software). Haplotype association analysis was carried out using THESIAS [23] and PHASE version [24]. Significant haplotype data was adjusted for multiple comparisons using Bonferroni and the false discovery rate (FDR) estimated. *P* values are those given by PHASE. *P* values less than 0.01 were considered significant. Analysis was limited to the six most common haplotypes (frequency >1%) in all studies.

## Results

### Baseline characteristics

The baseline characteristics of the participants in the three studies are presented in Table 1. More than a third of the children in GENDAI were overweight with 9.5% being obese. Subjects were classified as obese, overweight and non-overweight according to the International Obesity Task Force [17]. Measures of blood pressure, insulin, triglycerides, height and insulin resistance were significantly higher (*P* < 0.0001) and high density lipoprotein cholesterol significantly lower (*P* < 0.0001) in the overweight and obese group compared to their normal weight counterparts (data not shown). The young men in EARSII were all of a normal BMI, mean 23.10 kg/m^2^ 95% confidence intervals (CI) 22.91, 23.29. There were no differences in baseline measures between the offspring of the ‘cases’ and ‘controls’ with the exception of total cholesterol levels which were significantly higher in the cases (*P* < 0.001). The women in GrOW were mostly overweight (42.1%) and obese (34.7%), but were free of diabetes and CVD.

## Allele and haplotype frequencies in GENDAI, EARSII and GrOW

**Figure 1:**
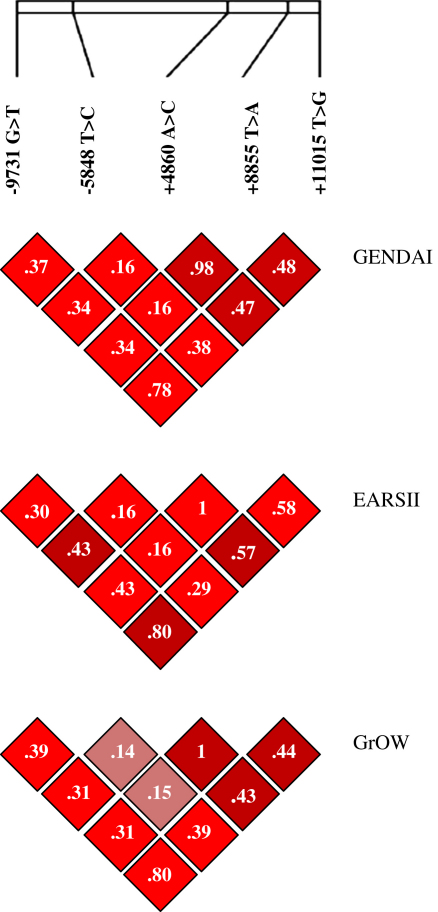
Linkage disequilibrium between the five *IL18* tagging single nucleotide polymorphisms in the Gene-Diet Attica Investigation on childhood obesity (GENDAI), European Atherosclerosis Research case control Study (EARSII) and the Greek Obese Women (GrOW). Single nucleotide polymorphism order; −9731 G > T (rs1946519), −5848 T > C (rs2043055), +4860 A > C (rs549908), +8855 T > A (rs360729), +11015 T > G (rs3882891). Colour represents D' - D'.95-1; - D'.80-.94; - D' –.75-.79. Values represent *r*^2^.

Genotypes for all five SNPs were determined in all studies and all genotypes were in HWE. In GENDAI there were no allele frequency differences between boys and girls or between normal weight children and their overweight and obese counterparts for any of the *IL18* variants. Similarly, in EARSII allele frequencies showed no ‘case’ ‘controls’ difference (data not shown). The genotypes and minor allele frequencies (MAF) for the *IL18* variants are shown in Appendices Table 1. High LD was observed between the five tSNPs in all three studies. D' values were between 0.75 and 1 and *r*^2^ values between 0.14 and 1 (Fig. 1).

Haplotypes were inferred by PHASE separately in all study groups. In total, six common haplotypes were observed and their frequencies are shown in Table 2. The rank order of haplotype frequencies were not the same for the three studies and frequencies varied significantly between the two Greek cohorts, GENDAI and GrOW (Global *P* = 0.006). There is also a significant difference in the frequencies between EARSII and GENDAI (Global *P* = 0.001) and EARSII and GrOW (Global *P* < 0.0001).

## IL18 univariate and haplotype associations with baseline and post-prandial measures

We investigated the effect of *IL18* variants on intermediate phenotypes of the metabolic syndrome in each of the three studies. The effect on post-prandial measures following an OFTT and OGTT in EARSII were also examined. tSNPs rs549908 and rs360729 are in complete LD with functional promoter variant rs187238. tSNPs rs1946519 and 3882891 are in complete LD with the 3′ UTR functional variant rs5744292 [16]. rs2043055 was not in LD with either of the functional SNPs.

### GENDAI

Apart from a modest association with rs2043055 on SBP and DBP (Appendices Table 2), no associations were statistically significant in the combined haplotype analysis with any baseline measures in the total sample (Table 3) or when the cohort was dichotomised by BMI (Data not shown).

### EARSII

rs2043055 was significantly associated with peak and area under the curve (AUC) triglycerides after an OFTT in the subjects classified as ‘cases’ (*P* = 0.001 for both). Homozygotes of the less frequent C allele had 17.63 mg/dl lower peak triglycerides and 0.7 less AUC compared to common T allele homozygotes. The same trend was observed in the ‘controls’ but did not reach statistical significance (Table 4). There was no interaction of case: control status with rs2043055 and no effect on post-prandial insulin and glucose. There was no association of rs2043055 with post-prandial measures after an OGTT (Data not shown). No associations were observed at the haplotypic level (Table 3).

### GrOW

rs2043055 C allele homozygotes exhibited higher insulin levels (*P* = 0.054), a higher HOMA_-IR_ estimate (*P* = 0.035) and a lower QUICKI estimate (*P* = 0.048) in comparison to carriers for the common T allele (Appendices Table 3). However, these associations did not reach statistical significance.

Highly significant associations were observed after haploytpe analysis with plasma insulin levels, HOMA_-IR_, HOMA-_β-cell_, and QUICKI estimates (Global *P* < 0.0001 for all associations) (Table 3). After further analysis and Bonferroni correction, it was observed that the phenotypic mean for insulin, HOMA_-IR_ and HOMA-_β-cell_ was significantly higher for Hap6 in comparison to the common haplotype, Hap1 (Bonferroni *P* < 0.001 for all associations). Insulin levels were 5.56 μIU/ml higher; HOMA_-IR_ and HOMA-_β-cell_ estimates were 1.34 and 20.75 higher, respectively. Furthermore, QUICKI was significantly lower in Hap6 in comparison to Hap1 (Bonferroni *P* < 0.001). The FDR for these associations was between 0.001 and 0.003% (*q*-value 0.001–0.003).

## Discussion

The major findings from this study are the effects of variation within *IL-18* using combined haplotypes analysis, on insulin levels and estimates of insulin resistance. Furthermore, this is the first report of the influence of a variation within *IL18* on post-prandial triglyceride levels, supporting the idea of IL-18 playing a role in metabolic processes.

Examining the SNPs individually, by univariate analysis in all three studies, associations were seen only with rs2043055. In EARSII there was a significant association of rs2043055 with peak and AUC triglycerides after an OFTT in the subjects classified as cases in EARSII. Homozygotes of the less frequent C allele had significantly lower peak triglycerides and smaller AUC compared to T allele homozygotes. These results suggest carriers of the C allele clear or absorb triglycerides faster than TT individuals and support the idea of IL-18 playing a role in metabolic processes. Post-prandial measures were not available in the GrOW study and therefore the associations observed in EARSII could not be replicated. However, there have previous reports of rs2043055 and its potential functionality as the variant distinguishes haplotypes associated with BMI [15] and IL-18 levels in healthy men and coronary artery bypass graft patients [25].

Considering the combined effect of the SNPs in haplotype analysis, six common haplotypes were observed, although frequencies varied significantly between the three studies. The allele frequencies and overall patterns of LD were similar in these studies yet the haplotype frequency differed, even among the two Greek studies. Haplotype diversity can have implications for designing association studies from genetically distinct populations and there is evidence in EARSII of allele frequency differences across Europe [26]. We have no explanation for this difference in haplotype frequency but it may reflect variation in selection criteria.

The haploytpe analysis in the 2 younger groups, GENDAI and EARSII, showed no association with intermediate traits. However in GrOW, one haplotype, Hap6, was associated with effects on insulin levels and estimates of insulin resistance and sensitivity. Compared to Hap1, Hap6 that was associated with higher plasma insulin levels and higher estimates of HOMA_-IR_ and HOMA-_β-cell_ in the GrOW study. The HOMA model is used to give an estimate of insulin sensitivity and ß-cell function from fasting plasma insulin and glucose concentrations [20]. The associations observed in GrOW suggest that those women who carried the Hap6 haplotype showed some insulin resistance, since as they had higher plasma insulin, but their glucose levels do not differ significantly from the other haplotypes. This is further supported by the QUICKI estimate of insulin sensitivity which is significantly lower in those with Hap6. QUICKI estimates have been shown to be lower in those who are overweight and diabetic when compared to non-obese and non-diabetic individulas [22]. It also appears that Hap6 carriers are yet to develop β–cell failure as their HOMA-_β-cell_ estimate is higher than those with Hap1.

Hap6 is relatively uncommon in GrOW (Frequency 2.6%). For this reason we repeated the haplotype analyses using the THESIAS program and utilised a bootstrap approach to take the uncertainty of inferring haplotypes into account, although studies have suggested that correcting or adjusting for uncertainty has little effect with inferred haplotypes [27]. All associations remained significant when repeated. A recent study of non-diabetic Caucasians reported the association of promoter variant, +183A > G (rs5744292), which is in complete LD with rs1946519 and rs3882891, with increased levels of serum IL-18, increased risk of metabolic syndrome and impaired insulin sensitivity [16]. Insulin sensitivity was significantly higher in subjects carrying the G allele and circulating IL-18 levels significantly lower. The C allele of the −137 G > C polymorphism (rs187238), which is in complete LD with rs549908 and rs360729, in the promoter region has also been associated with lower transcriptional activity [28]. The effect of variants on IL-18 levels and their association with insulin levels and sensitivity suggests that insulin sensitivity and metabolic syndrome may have a genetic basis [16].

*IL18* haplotypic effects on BMI have been reported in T2D and in subjects undergoing coronary artery bypass surgery [15]. However, in a healthy cohort of 3012 middle aged men single SNP and haplotype analysis with five *IL18* tSNPs showed no effect on BMI. There is an apparent absence of effect of *IL18* variation on BMI within all three of our studies. Bodyweight differences were only seen in the mouse *il-18* knock-out model in comparison to their wild-type littermates after six months of age and older [13]. Thus the effect *IL18* may only become apparent as subjects age and therefore the lack of effect in GENDAI and EARSII is not unexpected. It would appear the lack of association in GrOW may be due the study population, as those with a BMI over 30 are over represented, and power was limited. Furthermore, the participants in GrOW, although many were overweight, they were healthy. This is unlike the diseased cohorts which have reported the effect on BMI [15]. It is possible that the effects of IL-18 are exacerbated by disease.

Data presented on the *il18* knockout mouse suggested that il-18 was a satiety factor and was likely to be exerting its effect on the hypothalamus. Therefore, it seems possible that the IL-18 effect on BMI and metabolic syndrome may result through two distinct pathways. With a potential causal role in atherogenesis as well as T2D, IL-18 may be implicated in a number of complex diseases and their risk prediction. Tiret et al. [29] highlighted the role of *IL18* in cardiovascular disease, demonstrating that *IL18* haplotypes were associated with variation in IL-18 serum levels and cardiovascular mortality. These associations have been confirmed in a number of cohorts [15,25]. Markers of inflammation are significantly higher in those who are overweight in comparison to those of a normal weight and the mechanism whereby genetic variation of *IL18* is involved in the development of diabetes and metabolic syndrome is likely to be affected by inflammation and activated innate immunity [30,31].

In conclusion, the association of genetic variation within *IL18* on insulin levels and estimates of insulin resistance were only observed in our older GrOW study, suggesting that the effects of IL-18 appear to be more prominent as we age. Furthermore, the association of *IL18* variants with post-prandial measures provide support for IL-18 as a metabolic factor.

## Conflict of interest

There are no conflicts of interest.

## Figures and Tables

**Table 1:** Baseline characteristics of the Gene-Diet Attica Investigation on childhood obesity (GENDAI), European Atherosclerosis Research case control Study (EARSII) and Greek Obese Women study (GrOW).

	GENDAI (*n* = 882)		EARSII (*n* = 822)		GrOW (*n* = 349)		*P* value		
Mean	95%CI	Mean	95%CI	Mean	95%CI	p1	p2	p3
BMI (kg/m^2^)	19.92	(19.70, 20.14)	23.10	(22.91, 23.29)	28.45	(27.90, 29.00)	<0.001	<0.001	<0.001
Age (years)	11.2	(11.11, 11.20)	22.87	(22.68, 23.06)	46.95	(45.68, 48.23)	<0.001	<0.001	<0.001
Cholesterol (mg/dl)	185.38	(183.36, 187.41)	185.38	(183.36, 187.41)	214.59	(210.09, 219.18)	<0.001	<0.001	<0.001
Triglycerides (mg/dl)	60.57	(59.24, 61.94)	79.77	(77.76, 81.82)	86.71	(82.95, 90.66)	0.008	<0.001	<0.001
HDL-cholesterol (mg/dl)	51.73	(51.01, 52.46)	45.10	(44.50, 45.71)	50.68	(49.58, 51.82)	<0.001	0.123	<0.001
LDL-cholesterol (mg/dl)	119.63	(118.01, 121.29)	103.96	(101.98, 105.98)	143.63	(139.98, 147.37)	<0.001	<0.001	<0.001
Glucose (mg/dl)	84.97	(84.35, 85.59)	93.26	(92.71, 93.81)	94.46	(93.34, 95.60)	0.034	<0.001	<0.001
Insulin (μIU/ml)	6.83	(6.57, 7.10)	10.41	(9.95, 10.90)	7.83	(7.48, 8.20)	<0.001	<0.001	<0.001
HOMA_-IR_	1.43	(1.38, 1.49)	2.41	(2.29, 2.53)	1.83	(1.74, 1.92)	<0.001	<0.001	<0.001
HOMA_-β cell_	24.64	(23.57, 25.75)	127.70	(121.81, 133.86)	26.00	(24.72, 27.35)	<0.001	0.163	<0.001
QUICKI	0.363	(0.361, 0.366)	0.250	(0.247, 0.252)	0.349	(0.347, 0.352)	<0.001	<0.001	<0.001
Systolic Blood Pressure (mm/Hg)	119.70	(118.70, 120.72)	116.90	(116.16, 117.63)	–	–	<0.001	–	–
Diastolic Blood Pressure (mm/Hg)	74.02	(73.16, 74.89)	72.80	(72.09, 73.52)	–	–	0.035	–	–

% Male	47.4		100		0		<0.001	<0.001	<0.001
% Current Smokers	–		26		36		–	–	<0.01

Values are expressed as mean (±95% confidence intervals). p1 – GENDAI vs. EARSII, p2 – GENDAI vs. GrOW, p3 – EARSII vs. GrOW.

HDL-cholesterol – High density lipoprotein cholesterol; LDL-cholesterol – Low density lipoprotein cholesterol. Insulin resistance (IR) and β-cell function were estimated using the homeostasis model assessment (HOMA) with the following formulas: HOMA_-IR_ = fasting insulin (μIU/ml) x fasting glucose (mmol/l)/22.5, HOMA _β-cell_ = fasting insulin (μIU/ml) × 20/fasting glucose (mmol/l) − 3.5, quantitative insulin sensitivity check index (QUICKI) = 1/(log(fasting insulin (μIU/ml)) + log(fasting glucose (mg/dl)).

**Table 2:** Haplotype frequencies in the Gene-Diet Attica Investigation on childhood obesity (GENDAI), European Atherosclerosis Research case control Study (EARSII) and Greek Obese Women study (GrOW).

Haplotype		*IL18* -9731	*IL18* -5848	*IL18* + 4860	*IL18* + 8855	*IL18* + 11015	GENDAI		EARSII		GrOW		*P* value		
		*n*	Frequency	*n*	Frequency	*n*	Frequency	p1	p2	p3
Hap 1	12111	G	C	A	T	T	669	38.3	442	32.5	232	33.2	0.001	0.02	0.67
Hap 2	21222	T	T	C	A	G	401	22.9	363	26.5	136	19.5	0.02	0.06	<0.0001
Hap 3	11111	G	T	A	T	T	358	20.5	325	23.6	171	24.5	0.03	0.03	0.71
Hap 4	21112	T	T	A	T	G	251	14.4	169	12.3	124	17.8	0.10	0.04	0.001
Hap 5	22111	T	C	A	T	T	29	1.7	24	1.8	12	1.7	0.65	0.92	0.23
Hap 6	12222	G	C	C	A	G	27	1.5	35	2.3	18	2.6	0.08	0.09	0.21
Global *P* value													0.001	0.006	<0.0001

Haplotypes were inferred using PHASE. 1 – indicates common allele, 2 – rare allele.

Single nucleotide polymorphism order; −9731 G > T (rs1946519), −5848 T > C (rs2043055), +4860 A > C (rs549908), +8855 T > A (rs360729), +11015 T > G (rs3882891).

p1 GENDAI vs. EARSII; p2 GENDAI vs. GrOW; p3 EARSII vs. GrOW.

**Table 3:** Haplotypic mean for baseline and post-prandial measures in the Gene-Diet Attica Investigation on childhood obesity (GENDAI), European Atherosclerosis Research case control Study (EARSII) and Greek Obese Women (GrOW).

	n	BMI (kg/m^2^)		Triglycerides (mg/dl)		Insulin (μIU/ml)		Glucose (mg/dl)		HOMA_-IR_		HOMA _β-cell_		QUICKI	
Mean	SE	Mean	SE	Mean	SE	Mean	SE	Mean	SE	Mean	SE	Mean	SE
*GENDAI*															
Hap1 (12111)	669	20.13	0.13	65.24	0.86	7.96	0.19	85.50	0.37	1.73	0.05	29.57	0.73	0.37	0.00
Hap2 (21222)	401	20.10	0.17	63.91	1.12	7.92	0.25	85.18	0.47	1.69	0.06	29.94	0.95	0.37	0.00
Hap3 (11111)	358	20.30	0.18	63.19	1.17	8.11	0.25	85.52	0.50	1.73	0.07	30.39	0.99	0.37	0.00
Hap4 (21112)	251	20.56	0.21	63.40	1.39	8.02	0.30	86.22	0.59	1.73	0.08	29.99	1.18	0.36	0.00
Hap5 (22111)	29	21.23	0.63	60.60	4.08	8.18	0.88	85.87	1.73	1.74	0.23	31.11	3.43	0.37	0.01
Hap6 (12222)	27	19.53	0.65	62.10	4.22	7.48	0.91	84.08	1.79	1.58	0.24	28.52	3.55	0.37	0.01
*Global P* value		0.191		0.605		0.983		0.759		0.982		0.981		0.821	

*EARSII*															
Hap1 (12111)	442	22.51	0.4	71.75	3.94	12.93	0.64	93.53	1.08	2.96	0.16	145.73	14.57	0.26	0.06
Hap2 (21222)	363	22.32	0.4	69.35	4.34	12.13	0.58	93.21	1.06	2.81	0.14	132.13	10.57	0.24	0.03
Hap3 (11111)	325	22.00	0.4	70.38	4.48	13.25	0.69	95.56	1.06	3.16	0.17	151.01	15.93	0.28	0.04
Hap4 (21112)	169	22.26	0.5	59.04	5.51	12.00	0.97	93.22	1.47	2.77	0.24	142.95	21.55	0.27	0.07
Hap5 (22111)	24	22.60	1.1	109.52	9.84	15.53	2.87	99.03	3.02	3.88	0.71	168.66	72.80	0.26	0.46
Hap6 (12222)	35	22.55	1.1	55.94	15.81	12.51	2.66	92.93	3.89	2.89	0.62	161.77	99.15	0.29	0.29
*Global P* value		0.84		0.458		0.495		0.202		0.247		0.792		0.69	

*GrOW*															
Hap1 (12111)	232	29.42	0.67	95.24	5.60	7.82	0.53	95.25	1.24	1.87	0.13	26.01	2.04	0.35	0.00
Hap2 (21222)	136	29.65	0.71	94.01	5.93	7.44	0.56	94.89	1.32	1.77	0.14	24.71	2.16	0.36	0.00
Hap3 (11111)	171	28.63	0.72	97.41	5.97	7.95	0.56	93.18	1.32	1.85	0.14	27.36	2.17	0.35	0.00
Hap4 (21112)	124	28.81	0.77	97.16	6.36	7.43	0.60	93.78	1.41	1.74	0.15	25.14	2.32	0.36	0.00
Hap5 (22111)	12	27.98	1.71	105.60	14.21	8.75	1.33	95.73	3.15	2.09	0.33	29.53	5.17	0.35	0.01
Hap6 (12222)	18	29.34	1.41	87.68	11.67	13.38	1.10	95.64	2.59	3.21	0.27	46.76	4.25	0.33	0.01
*Global P* value		0.533		0.892		<0.001		0.436		<0.001		<0.001		0.001	
*Bonferroni P value*						<0.001				<0.001		<0.001		<0.001	

Data are presented as mean (standard error).

Single nucleotide polymorphism order; −9731 G > T (rs1946519), −5848 T > C (rs2043055), +4860 A > C (rs549908), +8855 T > A (rs360729), +11015 T > G (rs3882891).

GENDAI; all blood measures are adjusted for height, age, gender and BMI and anthropometric measures are adjusted for height, age, gender and mean Tanner score; Tanner stage (an estimate of pubertal status) was combined into one variable from the two Tanner measures to produce a mean Tanner score.

EARSII; all blood measures are adjusted for age, region, smoking status and BMI and anthropometric measures are adjusted for age, region and smoking status.

GrOW; all blood measures are adjusted for age, estrogen use, smoking status, menopausal status and body fat % and anthropometric measures are adjusted for age, estrogen use, smoking status and menopausal status.

Insulin resistance (IR) and β-cell function were estimated using the homeostasis model assessment (HOMA) with the following formulas: HOMA_-IR_ = fasting insulin (μIU/ml) × fasting glucose (mmol/l)/22.5, HOMA_-β-cell_ = fasting insulin (μIU/ml) × 20/fasting glucose (mmol/l) − 3.5, quantitative insulin sensitivity check index (QUICKI) = 1/(log(fasting insulin (μIU/ml)) + log(fasting glucose (mg/dl)).

**Table 4:** A. Effect of *IL18* -5848 variant on peak triglycerides after an oral fat tolerance test by case-control status in the European Atherosclerosis Research case control Study (EARSII). B. Effect of *IL18* -5848 variant on the area under the curve of triglycerides after an oral fat tolerance test by case-control status in EARSII.

*IL18* -5848 Genotype	Controls		Cases	
	*n*	Mean (SE)	*n*	Mean (SE)
A	Peak Triglycerides (mg/dl)			
TT	149	95.57 (3.63)	122	100.24 (4.19)
TC	161	95.68 (3.54)	160	93.13 (3.35)
CC	45	85.58 (5.99)	48	74.79 (4.97)
		*p* = 0.294		***p* = 0.001**

B	Area under the curve for Triglycerides			
TT	160	3.669 (0.157)	132	3.956 (0.167)
TC	167	3.516 (0.146)	170	3.606 (0.139)
CC	52	3.291 (0.272)	49	2.993 (0.232)
		*p* = 0.222		***p* = 0.002**

Data are presented as mean (standard error) by *IL18* -5848 T > C genotype (rs2043055). Adjusted for age, region, smoking status, BMI and fasting plasma triglyceride levels.

## Appendices Supplementary data

Appendices Table 1 *IL18* genotype and minor allele frequencies (MAF) in the Gene-Diet Attica Investigation on childhood obesity (GENDAI), European Atherosclerosis Research case control Study (EARSII) and Greek Obese Women study (GrOW).

Appendices Table 2 Association of *IL18* -5848 T > C variant with baseline plasma and anthropometric measures in Gene-Diet Attica Investigation on childhood obesity (GENDAI).

Appendices Table 3 Association of *IL18 -*5848 T > C variant with plasma and anthropometric measures in the Greek Obese Women Study.
