# Supplementary material for: Genetic variation within IL18 is associated with insulin levels, insulin resistance and postprandial measures
Source: Nutr Metab Cardiovasc Dis. 2011 Jul;21(7):476–84. doi: 10.1016/j.numecd.2009.12.004 (PMC3158674; doi:10.1016/j.numecd.2009.12.004)
Supplement: Appendices Table 1 — IL18 genotype and minor allele frequencies (MAF) in the Gene-Diet Attica Investigation on childhood obesity (GENDAI), European Atherosclerosis Research case control Study (EARSII) and Greek Obese Women study (GrOW). [file mmc1.doc]

Appendices Table 1. *IL18* genotype and minor allele frequencies (MAF) in the Gene-Diet Attica Investigation on childhood obesity (GENDAI), European Atherosclerosis Research case control Study (EARSII) and Greek Obese Women study (GrOW).

| **Variant** | **rs number** | **Genotype** | **GENDAI** | | |  | **EARSII** | | |  | **GrOW** | | |  | ***P* value** | | |
| --- | --- | --- | --- | --- | --- | --- | --- | --- | --- | --- | --- | --- | --- | --- | --- | --- | --- |
|  |  |  | **n** | **%** | **MAF (95% CI)** |  | **n** | **%** | **MAF (95% CI)** |  | **n** | **%** | **MAF (95% CI)** |  | **p1** | **p2** | **p3** |
|  |  |  |  |  |  |  |  |  |  |  |  |  |  |  |  |  |  |
| **-9731 G>T** | **rs1946519** | **GG** | 174 | 30.4 |  |  | 254 | 34.6 |  |  | 99 | 29.4 |  |  |  |  |  |
|  |  | **GT** | 285 | 49.8 |  |  | 363 | 49.5 |  |  | 167 | 49.5 |  |  |  |  |  |
|  |  | **TT** | 113 | 19.8 | 0.45 (0.42, 0.48) |  | 117 | 15.9 | 0.41 (0.38, 0.43) |  | 71 | 21.1 | 0.46 (0.42, 0.50) |  | 0.04 | 0.63 | 0.02 |
|  |  |  |  |  |  |  |  |  |  |  |  |  |  |  |  |  |  |
| **-5848 T>C** | **rs2043055** | **TT** | 239 | 37.1 |  |  | 292 | 40.0 |  |  | 134 | 40.1 |  |  |  |  |  |
|  |  | **TC** | 307 | 47.7 |  |  | 337 | 46.2 |  |  | 153 | 45.8 |  |  |  |  |  |
|  |  | **CC** | 98 | 15.2 | 0.39 (0.36, 0.42) |  | 101 | 13.8 | 0.37 (0.34, 0.39) |  | 47 | 14.1 | 0.37 (0.33, 0.41) |  | 0.25 | 0.37 | 0.98 |
|  |  |  |  |  |  |  |  |  |  |  |  |  |  |  |  |  |  |
| **+4860 A>C** | **rs549908** | **AA** | 322 | 51.6 |  |  | 353 | 47.8 |  |  | 172 | 51.5 |  |  |  |  |  |
|  |  | **AC** | 257 | 41.2 |  |  | 332 | 44.9 |  |  | 138 | 41.3 |  |  |  |  |  |
|  |  | **CC** | 45 | 7.2 | 0.28 (0.25, 0.30) |  | 54 | 7.3 | 0.30 (0.27, 0.32) |  | 24 | 7.2 | 0.28 (0.24, 0.31) |  | 0.26 | 0.99 | 0.36 |
|  |  |  |  |  |  |  |  |  |  |  |  |  |  |  |  |  |  |
| **+8855 T>A** | **rs360729** | **TT** | 319 | 50.4 |  |  | 358 | 48.4 |  |  | 170 | 51.5 |  |  |  |  |  |
|  |  | **TA** | 268 | 42.3 |  |  | 328 | 44.4 |  |  | 137 | 41.5 |  |  |  |  |  |
|  |  | **AA** | 46 | 7.3 | 0.28 (0.26, 0.31) |  | 53 | 7.2 | 0.29 (0.27, 0.32) |  | 23 | 7.0 | 0.28 (0.24, 0.31) |  | 0.59 | 0.74 | 0.44 |
|  |  |  |  |  |  |  |  |  |  |  |  |  |  |  |  |  |  |
| **+11015 T>G** | **rs3882891** | **TT** | 186 | 18.9 |  |  | 235 | 31.6 |  |  | 93 | 27.7 |  |  |  |  |  |
|  |  | **TG** | 328 | 51.7 |  |  | 384 | 51.7 |  |  | 170 | 50.6 |  |  |  |  |  |
|  |  | **GG** | 120 | 29.4 | 0.45 (0.42, 0.48) |  | 124 | 16.7 | 0.43 (0.40, 0.45) |  | 73 | 21.7 | 0.47 (0.43, 0.51) |  | 0.23 | 0.35 | 0.05 |

Data are presented as frequency (95% confidence intervals) by *IL18* genotype.

p1 GENDAI vs. EARSII; p2 GENDAI vs. GrOW; p3 EARSII vs. GrOW.
