# Supplementary material for: Genetic variation within IL18 is associated with insulin levels, insulin resistance and postprandial measures
Source: Nutr Metab Cardiovasc Dis. 2011 Jul;21(7):476–84. doi: 10.1016/j.numecd.2009.12.004 (PMC3158674; doi:10.1016/j.numecd.2009.12.004)
Supplement: Appendices Table 2 — Association of IL18 -5848 T > C variant with baseline plasma and anthropometric measures in Gene-Diet Attica Investigation on childhood obesity (GENDAI). [file mmc2.doc]

Appendices Table 2. Association of *IL18* -5848 T>C variant with baseline plasma and anthropometric measures in Gene-Diet Attica Investigation on childhood obesity (GENDAI).

|  |  | ***IL18* Genotype -5848 (rs2043055) minor allele frequency 0.39 (95% CI 0.36, 0.42)** | | | | | | | | | |
| --- | --- | --- | --- | --- | --- | --- | --- | --- | --- | --- | --- |
|  |  | **TT (n=239)** | |  | **TC (n=307)** | |  | **CC (n=98)** | |  |  |
|  |  | **Mean** | **(95% CI)** |  | **Mean** | **(95% CI)** |  | **Mean** | **(95% CI)** |  | ***P* Value** |
| **Triglycerides (mg/dl)** |  | 59.95 | (57.68, 62.31) |  | 60.25 | (58.21, 62.36) |  | 59.41 | (59.41, 67.05) |  | 0.342 |
| **LDL-cholesterol (mg/dl)** |  | 120.84 | (117.82, 123.93) |  | 117.76 | (115.15, 120.43) |  | 122.50 | (117.76, 127.43) |  | 0.144 |
| **HDL-cholesterol (mg/dl)** |  | 51.69 | (50.40, 53.01) |  | 51.63 | (50.49, 52.80) |  | 52.68 | (50.64, 54.79) |  | 0.669 |
| **Cholesterol (mg/dl)** |  | 186.25 | (182.58, 190.00) |  | 183.27 | (180.05, 186.55) |  | 189.95 | (184.12, 195.97) |  | 0.126 |
| **Insulin (µIU/ml)** |  | 7.08 | (6.65, 7.54) |  | 6.85 | (6.47, 7.24) |  | 6.62 | (6.00, 7.30) |  | 0.499 |
| **Glucose (mg/dl)** |  | 85.85 | (84.67, 87.04) |  | 84.21 | (83.18, 85.25) |  | 86.09 | (84.25, 87.97) |  | 0.067 |
| **HOMA_-IR_** |  | 1.51 | (1.41, 1.62) |  | 1.44 | (1.35, 1.53) |  | 1.42 | (1.28, 1.58) |  | 0.477 |
| **HOMA _β-cell_** |  | 25.74 | (23.97, 27.63) |  | 25.22 | (23.68, 26.87) |  | 23.59 | (21.12, 26.34) |  | 0.426 |
| **QUICKI** |  | 0.360 | (0.356, 0.364) |  | 0.363 | (0.360, 0.367) |  | 0.364 | (0.358, 0.370) |  | 0.428 |
| **Systolic Blood Pressure (mm/Hg)** |  | 118.70 | (116.86, 120.56) |  | 121.13 | (119.46, 122.81) |  | 116.13 | (113.54, 119.32) |  | 0.013 |
| **Diastolic Blood Pressure (mm/Hg)** |  | 73.30 | (71.70, 74.94) |  | 75.10 | (73.64, 76.59) |  | 70.87 | (68.42, 73.40) |  | 0.015 |
| **Weight (kg)** |  | 44.29 | (43.38, 45.21) |  | 43.98 | (43.17, 44.80) |  | 43.18 | (41.79, 44.61) |  | 0.470 |
| **BMI (kg/m^2^)** |  | 20.07 | (19.66, 20.49) |  | 19.99 | (19.63, 20.36) |  | 19.52 | (18.90, 20.17) |  | 0.376 |

Data are presented as mean (95% confidence intervals).

HDL-cholesterol - High density lipoprotein cholesterol**;** LDL-cholesterol - Low density lipoprotein cholesterol; Insulin resistance (IR) and β-cell function were estimated using the homeostasis model assessment (HOMA) with the following formulas: HOMA_-IR_ = fasting insulin (μIU/ml) x fasting glucose (mmol/l)/22.5, HOMA _β-cell_ = fasting insulin (μIU/ml) x 20 / fasting glucose (mmol/l) - 3.5, quantitative insulin sensitivity check index (QUICKI) = 1 / (log(fasting insulin (μIU/ml)) + log(fasting glucose (mg/dl)).

Tanner stage (an estimate of pubertal status) was combined into one variable from the two Tanner measures to produce a mean Tanner score.

All blood measures are adjusted for height, age, gender and BMI. All anthropometric measures are adjusted for height, age, gender and mean Tanner score.
