# Supplementary material for: Genetic variation within IL18 is associated with insulin levels, insulin resistance and postprandial measures
Source: Nutr Metab Cardiovasc Dis. 2011 Jul;21(7):476–84. doi: 10.1016/j.numecd.2009.12.004 (PMC3158674; doi:10.1016/j.numecd.2009.12.004)
Supplement: Appendices Table 3 — Association of IL18 -5848 T > C variant with plasma and anthropometric measures in the Greek Obese Women Study. [file mmc3.doc]

**Appendices Table 3.** Association of *IL18 -*5848 T>C variant with plasma and anthropometric measures in the Greek Obese Women Study.

|  |  | ***IL18* Genotype -5848 (rs2043055) minor allele frequency 0.37 (95% CI 0.33, 0.41)** | | | | | | | | | |
| --- | --- | --- | --- | --- | --- | --- | --- | --- | --- | --- | --- |
|  |  | **TT (n=134)** | |  | **TC (n=153)** | |  | **CC (n=47)** | |  |  |
|  |  | **Mean** | **(95% CI)** |  | **Mean** | **(95% CI)** |  | **Mean** | **(95% CI)** |  | ***P* Value** |
| **Triglycerides (mg/dl)** |  | 85.36 | (79.89, 91.22) |  | 87.88 | (82.58, 93.53) |  | 80.24 | (71.70, 89.80) |  | 0.378 |
| **LDL-cholesterol (mg/dl)** |  | 144.27 | (138.82, 149.96) |  | 141.38 | (136.35, 146.59) |  | 145.68 | (136.45, 155.54) |  | 0.642 |
| **HDL-cholesterol (mg/dl)** |  | 50.6 | (48.93, 52.33) |  | 50.35 | (48.79, 51.96) |  | 52.81 | (49.89, 55.90) |  | 0.343 |
| **Cholesterol (mg/dl)** |  | 214.9 | (208.32, 221.70) |  | 212.57 | (206.45, 218.97) |  | 216.17 | (205.05, 227.90) |  | 0.814 |
| **Insulin (µIU/ml)** |  | 7.62 | (7.13, 8.15) |  | 7.69 | (7.22, 8.19) |  | 8.91 | (7.95, 9.99) |  | 0.054 |
| **Glucose (mg/dl)** |  | 93.48 | (91.81, 95.18) |  | 94.42 | (92.83, 96.03) |  | 95.99 | (93.10, 98.98) |  | 0.328 |
| **HOMA_-IR_** |  | 1.76 | (1.64, 1.89) |  | 1.79 | (1.68, 1.92) |  | 2.11 | (1.87, 2.39) |  | 0.035 |
| **HOMA _β-cell_** |  | 25.59 | (23.72, 27.60) |  | 25.46 | (23.71, 27.33) |  | 29.55 | (25.98, 33.60) |  | 0.117 |
| **QUICKI** |  | 0.352 | (0.348, 0.356) |  | 0.351 | (0.348, 0.355) |  | 0.343 | (0.337, 0.349) |  | 0.048 |
| **Weight (kg)** |  | 72.02 | (69.72, 74.40) |  | 72.39 | (70.22, 74.63) |  | 74.92 | (70.91, 79.16) |  | 0.465 |
| **BMI (kg/m^2^)** |  | 28.49 | (27.61, 29.39) |  | 28.27 | (27.45, 29.11) |  | 29.29 | (27.78, 30.89) |  | 0.515 |

Data are presented as mean (95% confidence intervals).

HDL-cholesterol - High density lipoprotein cholesterol**;** LDL-cholesterol - Low density lipoprotein cholesterol; Insulin resistance (IR) and β-cell function were estimated using the homeostasis model assessment (HOMA) with the following formulas: HOMA_-IR_ = fasting insulin (μIU/ml) x fasting glucose (mmol/l)/22.5, HOMA _β-cell_ = fasting insulin (μIU/ml) x 20 / fasting glucose (mmol/l) - 3.5, quantitative insulin sensitivity check index (QUICKI) = 1 / (log(fasting insulin (μIU/ml)) + log(fasting glucose (mg/dl)).

All blood measures are adjusted for age, estrogen use, smoking status, menopausal status and body fat %.

All anthropometric measures are adjusted for age, estrogen use, smoking status and menopausal status.
