# Supplementary material for: Genetic variation within IL18 is associated with insulin levels, insulin resistance and postprandial measures
Source: Nutr Metab Cardiovasc Dis. 2011 Jul;21(7):476–84. doi: 10.1016/j.numecd.2009.12.004 (PMC3158674; doi:10.1016/j.numecd.2009.12.004)
Supplement: Supplementary file 4 [file mmc4.doc]

**Appendices information: EARSII Acknowledgements**

**EARS II Project Leader :**

D. St. J. O'Reilly, UK

**EARS II Project Management Group :**

F. Cambien, France

G. De Backer, Belgium

D. St. J. O'Reilly, UK

M. Rosseneu, Belgium

J. Shepherd, UK

L. Tiret, France

**The EARS II Group Collaborating Centres and their Associated Investigators :**

**Austria :** H. J. Menzel, Institute for Medical Biology and Genetics, University of Innsbruck, laboratory.

**Belgium :** G. De Backer, S. De Henauw, Department of Public Health, University of Ghent, recruitment centre.

**Belgium :** M. Rosseneu, Laboratorium voor Lipoproteïne Chemie/Vakgroep Biochemie, University of Ghent, laboratory.

**Denmark :** O. Faergeman, C. Gerdes, Medical Department I, Aarhus Amtssygehus, Aarhus, recruitment centre.

**Estonia :** M. Saava, K. Aasvee, Department of Nutrition and Metabolism, Estonian Institute of Cardiology, Tallinn, recruitment centre.

**Finland :** C. Ehnholm*, R. Elovainio**, J. Peräsalo, *National Public Health Institute, **The Finnish Student Health Service, Helsinki, recruitment centre.

**Finland** : Y.A. Kesäniemi*, M.J. Savolainen*, P. Palomaa**, *Department of Internal Medicine and Biocenter, Oulu, **The Finnish Student Health Service, University of Oulu, recruitment centre.

**France :** L. Tiret, V. Nicaud, O. Poirier, INSERM U525, Paris, EARS data centre, laboratory.

**France :** S. Visvikis, Centre de Médecine Préventive, Nancy, laboratory.

**France :** J. C. Fruchart, J. Dallongeville, Service de Recherche sur les Lipoprotéines et l'Athérosclérose (SERLIA), INSERM U325, Institut Pasteur, Lille, laboratory.

**Germany :** U. Beisiegel, C. Dingler, Medizinische Klinik Universitäts-Krankenhaus Eppendorf, Hamburg, recruitment centre and laboratory.

**Greece :** G. Tsitouris, N. Papageorgakis, G. Kolovou, Department of Cardiology, Evangelismos Hospital, Athens, recruitment centre.

**Italy :** E. Farinaro, Dept. of Medical Preventive Sciences, University "Frederico II" of Naples,

recruitment centre.

**The Netherlands :** L. M. Havekes, IVVO-TNO Health Research, Gaubius Institute, Leiden, laboratory.

**Portugal :** M. J. Halpern, J. Canena, Instituto Superior de Ciencas da Saude, Lisbon, recruitment centre.

**Spain :** L. Masana, J. Ribalta, A. Jammoul, A. LaVille, Unitat Recerca Lipids, University Rovira i Virgili, Reus, recruitment centre and laboratory.

**Switzerland :** F. Gutzwiller, B. Martin, Institute of Social and Preventive Medicine, University of Zurich, recruitment centre and laboratory.

**United Kingdom :** D. St J. O'Reilly, M. Murphy, Institute of Biochemistry, Royal Infirmary, Glasgow, recruitment centre and laboratory.

**United Kingdom :** S.E. Humphries, P.J. Talmud, V. Gudnason, R.M. Fisher, University College London School of Medicine, London, laboratory.

**United Kingdom :** D. Stansbie, A.P. Day, M. Edgar, Department of Chemical Pathology, Royal Infirmary, Bristol, recruitment centre and laboratory.

**United Kingdom :** F. Kee*, A. Evans**, *Northern Health and Social Services Board, **Department of Epidemiology and Public Health, the Queen's University of Belfast, Belfast, recruitment centre.
